# Treatment of Dropsy after Scarlet Fever

**Published:** 1849-12

**Authors:** S. Alison


*Treatment of Dropsy after Scarlet Fever.* By Dr. S. Alison.—For those cases of general dropsy in which the powers of life are considerable, in which the circulation is excited, and the kidneys are obviously congested, the perspiration of the skin is to be early secured, the bowels are to be purged, and blood is to be taken from the loins by cupping. The value of free evacuations from the alimentary canal was illustrated in a case in which severe vomiting and purging occurred, and in which, though the quantity of urine did not increase, the anasarca visibly declined.

When the disease of the kidney remains obstinate, and the strength will permit, a seton in the loins will form a most useful drain. Permanent fomentation of the loins with hot water will prove highly useful.

The examples of the disease which are associated with defective general power, whether from original defect of constitution, or the exhaustion of the eruptive disease, demand supporting measures. While the secretions are encouraged it will be necessary to give wine, vegetable tonics, and iron. Of the latter, the potassio-tartrate and the iodide are the best preparations. The employment of diuretics requires caution; the more stimulating of these being calculated, in active congestion of the kidney, to do much harm. When the renal affection has somewhat subsided, the author has found advantage from the use of spirits of nitre, iodide of potassium, and the alkaline diuretics.—*London Jour. of Medicine.*

